# Performance of the New ABC and MAP(ASH) Scores in the Prediction of Relevant Outcomes in Upper Gastrointestinal Bleeding

**DOI:** 10.3390/jcm12031085

**Published:** 2023-01-30

**Authors:** Rita Jimenez-Rosales, Jose Maria Lopez-Tobaruela, Manuel Lopez-Vico, Eva Julissa Ortega-Suazo, Juan Gabriel Martinez-Cara, Eduardo Redondo-Cerezo

**Affiliations:** 1 Department of Gastroenterology, “Virgen de las Nieves” University Hospital, Avenida de las Fuerzas Armadas 2, 18014 Granada, Spain; 2 University of Granada, 18010 Granada, Spain; 3 Department of Medicine, School of Medicine, University of Granada, 18016 Granada, Spain; 4 Biosanitary Institute of Granada (ibs.GRANADA), 18014 Granada, Spain

**Keywords:** upper gastrointestinal bleeding, mortality, intervention, risk score

## Abstract

*Background & Aims*: Several risk scores have been proposed for risk-stratification of patients with upper gastrointestinal bleeding. ABC score was found more accurate predicting mortality than AIMS65. MAP(ASH) is a simple, pre-endoscopy score with a great ability to predict intervention and mortality. The aim of this study was to compare ABC and MAP(ASH) discriminative ability for the prediction of mortality and intervention in UGIB. As a secondary aim we compared both scores with Glasgow-Blatchford score and AIMS65. *Methods*: Our study included patients admitted to the emergency room of Virgen de las Nieves University Hospital with UGIB (2017–2020). Information regarding clinical, biochemical tests and procedures was collected. Main outcomes were in-hospital mortality and a composite endpoint for intervention. *Results*: MAP(ASH) and ABC had similar AUROCs for mortality (0.79 vs. 0.80). For intervention, MAP(ASH) (AUROC = 0.75) and ABC (AUROC = 0.72) were also similar. Regarding rebleeding, AUROCs of MAP(ASH) and ABC were 0.67 and 0.61 respectively. No statistically differences were found in these outcomes. With a low threshold for MAP(ASH) ≤ 2, ABC and MAP(ASH) classified a similar proportion of patients as being at low risk of death (42% vs. 45.2%), with virtually no mortality under these thresholds. *Conclusions*: MAP(ASH) and ABC were similar for the prediction of relevant outcomes for UGIB, such as intervention, rebleeding and in-hospital mortality, with an accurate selection of low-risk patients. MAP(ASH) has the advantage of being easier to calculate even without the aid of electronic tools.

## 1. Introduction

Incidence of upper gastrointestinal bleeding (UGIB) has decreased in the last decades (from 60.7/100,000 in 1993/94 to 47.7/100,000 in 2000), probably due to advances in peptic ulcer treatment and Helicobacter pylori eradication. Despite this progress, it is still a significant cause of hospital admission, with considerable morbidity and mortality (up to 10% in some series) [1,2,3].

Many risk scores (RS) like AIMS65 [4], Progetto Nazionale Emorragia Digestiva (PNED) [5], full Rockall [6] or Glasgow Blatchford [7] have been proposed to predict outcomes and stratify patients according to their risk. However, they showed some lack of discriminative performance to predict mortality in patients with UGIB and some of them (like full Rockall score) require endoscopic information to be calculated, which have limited their clinical use. In 2020, Laursen et al. published a new pre-endoscopy risk score for upper and lower gastrointestinal bleeding, ABC score, based on age, blood tests and comorbidities [8] (Table 1). This score was better for the prediction of mortality than AIMS65 and PNED, which seemed to be the best previous scores for this outcome [4,5,9]. MAP(ASH) is a simple pre-endoscopy score published by Redondo-Cerezo et al. in 2019, created from a Spanish cohort of 547 patients and subsequently validated in an international one of 3012 patients [10]. MAP(ASH) score is a simple and easy to remember score, consisting in an acronym for altered mental status, ASA score, heart rate (pulse), albumin, systolic blood pressure and hemoglobin. It was designed as an easy and widely available tool to quickly classify patients with an increased risk of death. When compared with the other risk scores, it showed a great performance predicting intervention and mortality in UGIB, with the extra advantage of being easier to calculate [10] (Table 1).

In clinical practice, defining scores’ thresholds to stratify patients according to their risk of intervention and death would be useful to improve their management, avoiding low-risk patients’ admissions and prioritizing interventions for high-risk individuals. For ABC, authors determined a cut-off value of ≤3 for a very low risk of death, with 56% of patients included in this group and a mortality of 0.7% within 30 days [8], this threshold was established at ≤1 points for MAP(ASH) [10].

The main goal of our study was to compare the discrimination ability of ABC and MAP(ASH) scores to predict mortality and need for intervention in patients admitted to our hospital with upper gastrointestinal bleeding. Secondary aims were to evaluate performance of both scores to classify patients into low risk of death and intervention, as well as to compare both of them with other scores.

## 2. Methods

### 2.1. Study Design and Population

We performed a retrospective analysis on a prospective registry on consecutive and unselected patients admitted to the emergency room (ER) of Virgen de las Nieves University Hospital with UGIB (January 2017–December 2020).

Variceal and non-variceal etiologies were included, as well as patients already in hospital for another reason who developed UGIB. Patients who refused to sign the informed consent for the study were excluded. Patients were followed throughout hospitalization and six months after discharge.

### 2.2. Definitions

Upper GI hemorrhage was defined as bleeding from the upper GI tract manifested as melena and/or hematemesis (including coffee ground vomiting).

Rebleeding was defined as a new bleeding episode from the same source after a successful hemostasis, manifested as fresh hematemesis and/or melena associated with the development of shock (pulse > 100 beats/min, systolic blood pressure < 100 mmHg) or a reduction in hemoglobin concentration ≥2 g/dL over 24 h, after a successful endoscopic and clinically apparent hemostasis. Rebleeding also included cases requiring a second interventional endoscopy, or radiology, or surgery.

Need for intervention was defined by a composite endpoint that includes red blood cell transfusion, endoscopic treatment, interventional radiology or surgery. Along with mortality, this endpoint can be considered central, as selects patients who can be safely discharged from the ER.

### 2.3. Management

Most of the patients underwent endoscopy, and only patients with unstable medical or surgical conditions precluding it did not received endoscopy. The timing of the procedure, the hemostasis methods and the need for endoscopic therapy were determined by the on-duty gastroenterologist (all of them with extensive experience and skills in endoscopic hemostasis), always in accordance with current management guidelines. The procedure was performed in every case within 24 h after the admission or the in-hospital bleeding event in case the patient had been previously admitted for another condition. The need for transfusion was determined at the discretion of the treating physician, usually in the ER, following strict criteria as previously published, establishing a threshold of 7 g/dL in the average patient and 8 g/dL in those with high-risk heart disease [11,12]. Patients’ management was based on guidelines’ recommendations as follows: All patients received high-dose acid suppression therapy and, if variceal bleeding was suspected, treatment with somatostatine and antibiotics was prescribed. Endoscopic therapy was applied to patients with high-risk bleeding stigmata and consisted in injection therapy, thermal therapy or mechanical therapy (usually clipping), or two of those methods, but not adrenaline alone. Hemostatic powders were sometimes used as a recue method or in diffuse bleedings, such as in some neoplasms. In acute variceal bleeding band ligation, endoscopic sclerotherapy, tissue glue injection, or transjugular intrahepatic portosystemic shunt were used. Patients underwent surgery or interventional radiology if bleeding persisted despite of endoscopic therapy, or if rebleeding occurred after two therapeutic endoscopies [13,14,15,16,17].

### 2.4. Data Collection

Data on consecutive and unselected patients admitted to the emergency room with UGIB were collected. Information regarding patients’ demographic data, current medications (including antiplatelet, anticoagulants, steroidal and non-steroidal anti-inflammatory drugs), comorbidities, clinical presentation, hemodynamic parameters, admission laboratory test results and endoscopic findings was collected. Interventions, including the need for blood transfusion and the number of packed red blood cells units per patient, endoscopic therapy, interventional radiology guided hemostasis and surgery were registered. Clinical outcomes were in-hospital mortality, rebleeding, length of hospital stay, and delayed 6-months hemorrhagic and cardiovascular events and mortality. Outcomes were prospectively assessed and recorded by the investigators, directly when the patient was admitted and with direct phone calls and electronic charts consultations when he/she was discharged. The research team had access to our regional unified electronic chart, which includes hospital and primary care information, and it was used for the follow-up. When doubts arose from data, the researchers phoned the patients or their household relatives and tried to know about the main outcomes. Collected data were used to calculate the ABC score [8], MAP(ASH) score [10], Glasgow Blatchford Score (GBS) [7], AIMS65 score [4], and American Society of Anesthesiologists (ASA) score [18] at admission for each patient.

### 2.5. Data Analysis

Patients previously included in the validation of both MAP(ASH) and ABC scores were excluded from the analysis.

Statistical analysis was carried out using the software IBM SPSS Statistics 21.0. All tests were two sided and *p* < 0.05 was considered significant.

Continuous variables were expressed as medians.

Ability to predict mortality, rebleeding, need for endoscopic therapy and the composite endpoint for intervention among risk scores were evaluated by AUROCs. Comparisons of scores’ AUROCs were performed with the deLong test.

Comparisons between the different groups (ABC low risk vs. MAP(ASH) ≤ 1 low risk and MAP(ASH) ≤ 2 low risk) were performed by means of chi-square test. Sensitivity, specificity, PPV and NPV were calculated for MAP(ASH) and ABC in low-risk patients.

## 3. Results

### 3.1. Patients’ Characteristics

795 patients with upper gastrointestinal bleeding were recruited. Median age was 64.18 years (interquartile range 55–78) and 33.2% were females. Variceal bleeding was the cause of UGIB in 13.5% (78), whereas the main causes of non-variceal bleeding were duodenal ulcer (27%), gastric ulcers (21%), esophagitis (9.4%) and upper GI neoplasms (9%). Previously admitted patients comprised 16.6% of our cohort. Throughout the admission, 79 patients died (9.9%), 75.3% needed any type of intervention (therapeutic endoscopy, interventional radiology, surgical treatment or transfusions). Comorbidities were present in a majority of our patients (79.3%), being the main ones hypertension (49.2%), diabetes (27.8%), cirrhosis (20.3%) and atrial fibrillation (18.7%) (Table 2).

### 3.2. Predicting Ability of Pre-Endoscopy Risk Scores

**Figure 1:**
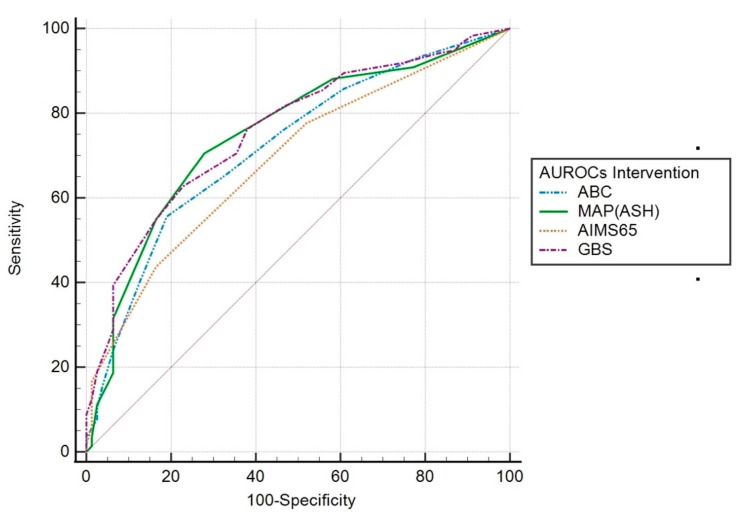
AUROC of the scoring systems for predicting intervention.

Intervention. Scoring systems AUROCs for this outcome were as follows: GBS 0.76 (95% CI 0.70–0.81; *p* < 0.001); MAP(ASH) 0.75 (95% CI 0.69–0.81; *p* < 0.001), ABC 0.72 (95% CI 0.66–0.78; *p* < 0.001); AIMS65 0.69 (95% CI 0.63–0.75; *p* < 0.001). The AUROC for GBS was significantly higher than that of the AIMS65 (*p* = 0.017). There was no significant difference between the other scoring systems (Figure 1).

**Figure 2:**
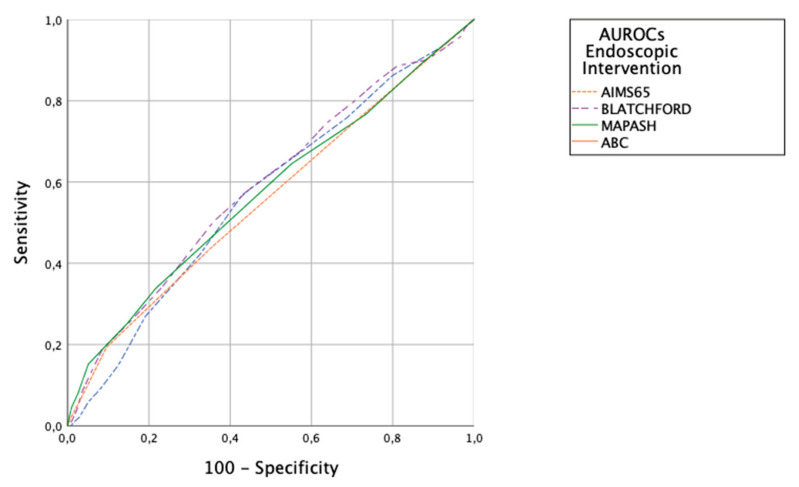
AUROCs of the scoring systems for predicting endoscopic interventions.

Endoscopic Intervention. Scoring prediction ability was poor, with AUROCs for MAP(ASH) 0.57 (95% CI 0.53–0.61), for ABC 0.57 (95% CI 0.53–0.61), GBS 0.59 (95% CI 0.55–0.63), AIMS65 0.56 (95% CI 0.52–0.60). There were no significant differences between the scores (Figure 2).

**Figure 3:**
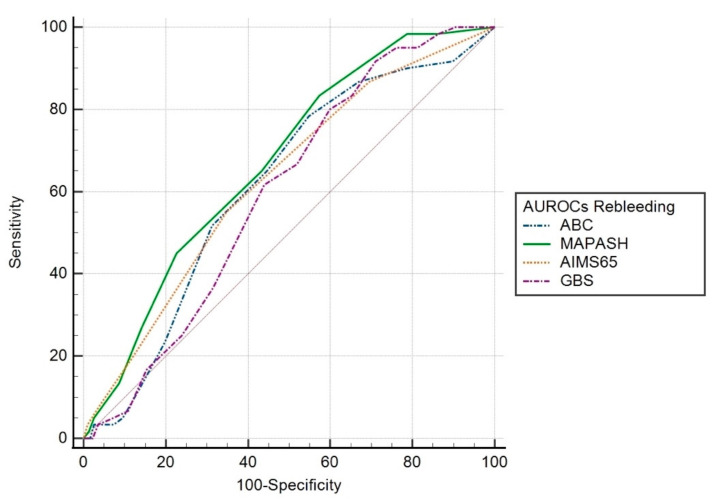
AUROC of the scoring systems for predicting rebleeding.

Rebleeding. Scoring AUROCs were as follows: MAP(ASH) 0.67 (95% CI 0.60–0.74; *p* < 0.001), AIMS65 0.63 (95% CI 0.56–0.70; *p* = 0.001), ABC 0.61 (95% CI 0.54–0.69; *p* = 0.005); GBS 0.60 (95% CI 0.53–0.66; *p* = 0.017). The AUROC for MAP(ASH) was significantly higher than that of the GBS (*p* = 0.026). There was no significant difference between the other scoring systems (Figure 3).

**Figure 4:**
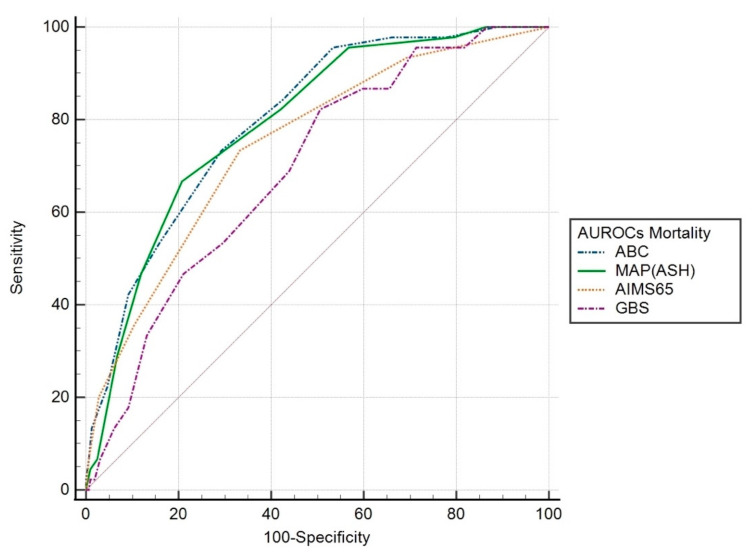
AUROC of the scoring systems for predicting in-hospital mortality.

In-hospital mortality. Scoring systems AUROCs were: ABC 0.80 (95% CI 0.74–0.86; *p* < 0.001), MAP(ASH) 0.79 (95% CI 0.73–0.86; *p* < 0.001), AIMS65 0.75 (95% CI 0.67–0.82; *p* < 0.001), GBS 0.69 (95% CI 0.62–0.77; *p* < 0.001). The AUROCs for MAP(ASH) and ABC were significantly higher than that of the GBS (*p* = 0.005 and *p* = 0.014, respectively). There was no significant difference between the other scoring systems (Figure 4).

### 3.3. Low-Risk Patients

Considering MAP(ASH) previously defined threshold for a very low risk of death (≤1 points), ABC score considered a higher proportion of patients in this group when compared to MAP(ASH) (42% vs. 28.3%), with similar mortality rate (0.5% vs. 0.9%).

For this reason, we evaluated the threshold MAP(ASH) ≤2 to define patients with low risk of death. With this cut-offs, ABC score and MAP(ASH) classified a similar proportion of patients as being “low risk” (42% vs. 45.2%), with a similar mortality rate (0.5% vs. 0.9%; *p* = 0.09). Only two patients died with a low-risk MAP(ASH), in both cases because of a non-related cause of death, which had been the main reason for admission for both patients, being upper GI bleeding an additional complication. One of these patients was the casualty identified as low-risk by ABC. For discharged patients, mortality was zero. In our cohort, other scores’ low-risk thresholds selected a very different proportion of patients, GBS ≤ 1 (3.4%) and AIMS65 ≤ 1 (60%), selected mortalities within the low-risk group of 0.1% and 3.4% respectively. Table 3 depicts sensitivity, specificity, PPV and NPV for MAP(ASH), ABC, GBS and AIMS65 in low-risk patients regarding mortality and need for intervention.

## 4. Discussion

Our results show that MAP(ASH), a risk score that can be easily calculated prior to endoscopy, in the emergency department, performs well in predicting relevant outcomes in UGIB: intervention, rebleeding and in-hospital mortality. It is comparable to ABC predicting mortality and to GBS regarding intervention, both scores previously identified as the best for these outcomes [8]. MAP(ASH) was also superior to the existing pre-endoscopy risk scores for the prediction of rebleeding. Our results suggest that MAP(ASH) is a risk-assessment tool for UGIB patients able to predict all outcomes of interest, in contrast with Laursen et al. recommendation about calculation of two scores for each patient to predict every relevant outcome [8]. Other authors [19] have recently recommended the use of up to three scores to assess patient’s risk, depending on the situation and, somehow redundantly, on the risk itself. Interestingly, this group included GBS and ABC but not MAP(ASH) in their comparisons. In this sense, we have observed that both MAP(ASH) and ABC can be used as single tools in this setting, which is both realistic regarding the widespread use of scores, and more convenient. Another report that included MAP(ASH) in the analysis observed similar results regarding ABC, although concerns about methodology, especially when calculating MAP(ASH) seem likely [20].

We also found that with the cut-off point originally defined to identify patients as low-risk of death for MAP(ASH) (≤1) few patients were identified as low-risk (half that for ABC), although mortality within this group was low. Shifting this cut-off point for a higher one (≤2) a greater proportion of patients were identified as low-risk (comparable to ABC), maintaining a similar death rate in this group. These results suggest that changing the cut-off point to identify patients with low-risk of death to MAP(ASH) ≤2 would improve its clinical relevance. Thresholds changes for different outcomes have been previously performed [21] and may be needed in ongoing research in order to establish the best cut-off point for the main outcomes. In this sense, our data show that low thresholds calculated for GBS [7] and AIMS65 [4] lack of clinical applicability, being GBS highly accurate excluding mortality but selecting a very small proportion of patients, offering little advantage sparing admissions as a too restrictive tool, and selecting AIMS65 up to 60% of patients as low-risk, but with a critical proportion of deaths among this low-risk group. Thus, both scores seem unfit for low-risk patients’ selection, the ones that could be directly discharged from the Emergency Department.

Our findings are consistent with those of Li et al., that showed that the largest AUC to predict 30-day mortality in UGIB was for ABC score (0.833) being MAP(ASH) similar (0.781), without statistically significant differences between both scores [22]. More, Laursen et al. [8] found that ABC score was closely associated with 30-day mortality in UGIB, performing better than AIMS65 score (0.81 vs. 0.65; *p* < 0.001) in the validation cohort, and better than all the scores included in the development cohort. These results have been confirmed in further reports by the same group [21]. However, these studies did not include MAP(ASH).

Regarding intervention, Li et al. found that MAP(ASH) was the score with the best AUROC for this event in UGIB (0.783), with no statistically significant differences with GBS, ABC, and AIMS65 [22]. In Saffouri et al. paper [21], GBS was the best predictor for intervention (considered as major transfusion and endoscopic therapy), which is consistent with our findings. In our analysis, GBS showed the best AUROC for this outcome, being MAP(ASH) similar, not finding statistically significant differences between both scores. The differences with Li et al. [22] may be explained by the fact that the cohort used by Li includes only patients older than 65 years. Considering low-risk patients’ selection, MAP(ASH) was the best tool for ruling out intervention, being the only score with a negative predictive value above 90% (Table 3). In the particular, but essential outcome of endoscopic intervention, no score seemed to be really accurate, in a consistent tendency with what has been published before [8,21]. In this setting, scores should be useful for the identification of high-risk patient, in need for early escalation to higher levels of care, including intensive care unit. Delayed escalation has been related with an increased mortality in these patients [23].

In other essential outcome such as rebleeding, our results were comparable to those of Li et al. [22]. In both studies MAP(ASH) was the score with the best AUROC to predict this outcome, without differences with AIMS65, ABC and GBS in this paper. By contrast, in our study, MAP(ASH) was statistically better than GBS. As mentioned for the need for intervention, this difference may be justified by the exclusion of patients under 65 in the study of Li et al. In this particular outcome, we should consider scores’ performance disappointing, with AUROCS below 0.7 in the different published reports [8,10,24], which can be otherwise reasonable because they were not designed for this outcome. Having recurrent bleeding has been recognized as a strong predictor for death [25,26], scores should be adapted for a better prediction ability in this particular outcome.

Considering low-risk patients, increasing MAP(ASH) cut off threshold to ≤2 helps to identify patients with UGIB who are at very low risk of death, and Li et al. found the same for older adults with UGIB, in which the best score cutoffs for predicting mortality were 3 or more for MAP(ASH) [22]. Previously, GBS was considered an adequate tool for the identification of low-risk individuals [7,9,13,27], however AIMS65 was not designed for low-risk patients detection, and this was not addressed in the original study [4]. Nevertheless, in subsequent studies it has showed some discrimination ability for this purpose [28]. In our series, MAP(ASH) ≤ 2 would have selected a wider proportion of patients for a safe discharge, with no deaths in this group, which makes the score highly appropriate for this purpose, quite similar to ABC, but with advantages over the other scores.

The main limitation of our research is the inclusion of patients from a single center that is also a referral one, which could reduce the applicability of the results. However, this limitation can be attenuated by the fact that, for some areas, ours is the primary emergency department, so that patients of different complexity have been included. Moreover, our hospital has 24/365 availability of endoscopy, and this could lead to earlier endoscopies and a higher rate of endoscopic therapy. Nevertheless, we always decide based on current practice guidelines. Furthermore, endoscopists experience might bias the results, but not every gastroenterologist who performed urgent endoscopies was a dedicated endoscopist, and our experience can be extensible to other centers. An important strength was the prospectively and systematic collection of data by the research team for our registry.

We included variceal and non-variceal bleeding because patients present to the emergency room with UGIB, usually with no prior endoscopy, and many times with no previous information suggesting a given etiology. We believe scores should be designed for the syndrome found in the clinical setting, and not for a given, not so many times known, etiology. This same idea has guided other groups when trying to build the best score for every type of gastrointestinal bleeding, such as ABC [8].

For similar reasons, we included patients who presented with UGIB while already inpatients.

Laursen et al. concluded in their study that two risk scores are needed for predicting the two important outcomes in UGIB, ABC score for mortality and GBS for intervention [8]. However, they did not include MAP(ASH) in their comparison. GBS and MAP(ASH) were similar predicting intervention, and our score was identical to ABC at predicting mortality. Considering our results, MAP(ASH) could be considered as a single pre-endoscopy score, easy to calculate, capable of adequately predict both outcomes, simplifying initial evaluation patients in the emergency room.

## 5. Conclusions

MAP(ASH) score is a pre-endoscopy risk score that can be used early after presentation in UGIB to estimate all outcomes of interest such as need for intervention, rebleeding and mortality. Its easy calculation, based on the possibility to recall its items very effortlessly in any clinical setting, with no need for an app or software, before endoscopy, makes it a very good tool for its widespread use in emergency rooms. ABC was also an excellent tool, although MAP(ASH) showed some subtle advantages, as a better performance when ruling out intervention in ‘low-risk’ patients. Moving the cut-off point to identify patients with low risk of death to ≤2 would improve its clinical usefulness, by increasing the proportion of patients identified as low risk who might be discharged from the ER, sparing admission.

## Figures and Tables

**Table 1:** MAP(ASH) and ABC score calculation.

**MAP(ASH) Score**	
**Risk Factor**	**Value**
M: Altered mental status (Glasgow < 15)	1
A: ASA score > 2	1
P (pulse): HR > 100	1
A: Albumin < 2.5 mg/dL	2
S: SBP < 90 mmHg	2
H: Hemoglobin < 10 g/dL	2
**ABC score**	
**Risk factor**	**Value**
**Age**	
60–74 years	1
≥75 years	2
**Blood tests**	
Urea > 10 mmol/L	1
Albumin < 3 g/dL	2
Creatinine	
100–150 μmol/L	1
>150 μmol/L	2
**Comorbidity**	
Altered mental status	2
Liver cirrhosis	2
Disseminated malignancy	4
ASA score	
3	1
≥4	3

**Table 2:** Characteristics of patients, treatment and outcomes.

	*n*	%
Male	534	67.2
Medical History		
Cirrhosis	161	20.3
Chronic lung diseases	89	11.2
Chronic renal disease	124	15.6
Heart failure	81	10.2
Myocardial infarction	94	11.8
Atrial fibrilation	149	18.7
Previous stroke	61	7.7
Previous GI bleeding events	191	24.1
Hypertension	391	49.2
Diabetes	221	27.8
Peripheral vascular disease	33	4.2
Neoplasm	111	14
Smoking habit	168	22.6
Alcoholic habit	154	21.2
Medications		
NSAIDS	159	20.4
Aspirin	169	21.3
Clopidogrel	27	3.4
Oral anticoagulants	176	22.1
Steroids	20	2.5
Immunosuppressants	15	2
Relevant variables and scores components		
Age (years)	64.18	Range: 14–94
Albumin (g/dL)	3.2	1.1–5.3
International normalized ratio	1.52	0.9–10.0
Systolic blood pressure	112	55–195
Pulse	89.5	37–144
Hemoglobin	9.5	3.2–18.1
Urea	84.1	9–135
Mental status change	63	8%
Findings at endoscopy		
Duodenal/Gastric Ulcer	368	46.4
Erosions	74	9.3
Esophagitis	78	9.8
Neoplasms	50	6.3
Esophageal varices	99	12.5
Active bleeding	212	26.7
Endoscopic intervention	371	46.8
Outcomes		
In-hospital mortality	79	9.9
Rebleeding	91	11.5
Surgery	39	3.8
Interventional radiology	14	1.8
Transfusion	320	55.4
Length of stay	9 ± 14.59	
Delayed cardiovascular events	46	6.1
Delayed hemorrhagic events	101	13.1
Delayed deaths	44	5.9
Scores (mean ± SD)		
AIMS65	1.28 ± 1.31	
Glasgow Blatchford	11.29 ± 4.44	
ABC	4.5 ± 2.83	
MAP(ASH)	3.27 ± 2.09	
ASA	2.63 ± 0.77	

**Table 3:** Discriminative abilities for in-hospital mortality and intervention in classified low-risk patients.

		**MAP(ASH)**	**ABC**	**G** **BS**	**AIMS65**
**In-hospital mortality**	Sensitivity	48.1% (95% CI 43.4–52.7%)	44.8% (95% CI 40–49.5%)	3.7% (95% CI 2.5–5.4%)	63.9% (95% CI 59–67%)
	Specificity	93.8% (95% CI 79.9–98.3%)	96.8% (95% CI 83.8–99.4%)	98.7% (95% CI 93.1–99.8%)	68.2% (95% CI 56–78%)
	PPV	99.1% (95% CI 96.7–99.7%)	99.5% (95% CI 97.2–99.9%)	96% (95% CI 80.5–99.3%)	94.3% (95% CI 91.5–96.2%)
	NPV	11.6% (95% CI 8.1–16.1%)	10.9% (95% CI 7.8–15%)	11% (95% CI 8.9–13.5%)	18.6% (95% CI 14.2–24%)
**Intervention**	Sensitivity	74.6% (95% CI 67–81%)	62.2% (95% CI 54–70%)	6.5% (95% CI 3.7–10.9%)	81% (95% CI 74–86%)
	Specificity	69.5% (95% CI 65.2–73.5%)	67.7% (95% CI 63.4–72%)	97.8% (95% CI 96.3–98.7%)	45.5% (95% CI 41–50%)
	PPV	42.4% (95% CI 36.4–48.6%)	36.8% (95% CI 31–43%)	48% (95% CI 30–66.5%)	31% (95% CI 26.4–35.4%)
	NPV	90.1% (95% CI: 86.6–92.8%)	85.6% (95% CI 82–89%)	77% (95% CI 74–80%)	88.8% (95% CI 84–92%)

NPV, negative predictive values; PPV, positive predictive values.

## Data Availability

Data, analytic methods, and study materials will not be made available to other researchers.
